# Effects of social restrictions on people with dementia and carers during the pre‐vaccine phase of the COVID‐19 pandemic: Experiences of IDEAL cohort participants

**DOI:** 10.1111/hsc.13863

**Published:** 2022-06-13

**Authors:** Claire Pentecost, Rachel Collins, Sally Stapley, Christina Victor, Catherine Quinn, Alexandra Hillman, Rachael Litherland, Louise Allan, Linda Clare

**Affiliations:** ^1^ REACH: The Centre for Research in Ageing and Cognitive Health, University of Exeter Medical School University of Exeter Exeter United Kingdom; ^2^ College of Health, Medicine and Life Sciences, Department of Health Sciences Brunel University London London UK; ^3^ Centre for Applied Dementia Studies University of Bradford Bradford UK; ^4^ Wolfson Centre for Applied Health Research Bradford United Kingdom; ^5^ School of Social Sciences Swansea University Swansea UK; ^6^ Innovations in Dementia CIC Exeter UK; ^7^ NIHR Applied Research Collaboration South‐West Peninsula Exeter UK

**Keywords:** Alzheimer's disease, carers, pandemic, qualitative analysis, vaccine

## Abstract

This qualitative study was designed to understand the impact of social distancing measures on people with dementia and carers living in the community in England and Wales during a period of social restrictions before the COVID‐19 vaccination roll‐out. We conducted 12 semi‐structured interviews with people with dementia aged 50–88 years, living alone or with a partner, and 10 carers aged 61–78 years, all living with the person with dementia. Three of the interviews were with dyads. Participants were recruited during November and December 2020. We used framework analysis to identify themes and elicit suggestions for potential solutions. We identified three interrelated themes. People with dementia experienced a *fear of decline* in capabilities or mood and attempted to mitigate this. Carers noticed changes in the person with dementia and increased caring responsibilities, and for some, a change in the relationship. Subsequently, reduced confidence in capabilities to navigate a new and hostile environment created a cyclical *dilemma of re‐engaging* where an inability to access usual activities made things worse. People with dementia and carers experienced *neglect* and being alone in their struggle, alongside feeling socially excluded during the pandemic, and there was little optimism associated with the upcoming vaccine programme. People found their own solutions to reduce the effects of isolation by keeping busy and being socially active, and practising skills deemed to help reduce the progression of dementia. This and some limited local public initiatives for the general public facilitated feelings of social inclusion. This study adds understanding to existing evidence about the longer‐term experience of social isolation several months into the pandemic. It highlights the importance of health and community groups and suggests how services can find ways to support, include, and interact with people with dementia and carers during and after social restrictions.

**What is known about this topic**

- Social restrictions to manage COVID‐19 infections had negative impacts on cognitive functioning and mood for people with dementia early in the pandemic.
- Carers struggled with the temporary closure of dementia services and associated respite.

**What this paper adds**

- People with dementia and carers did not feel they could return to previous activities when things reopened, and this did not change when the vaccination programme was announced.
- A further decrease in confidence and perceived decline in people with dementia was a barrier to returning to usual activities.
- People with dementia attempted to minimise decline and to stay socially engaged, but they, and their carers felt they did this alone, and felt socially excluded during the pandemic.

## 1 INTRODUCTION

The social distancing measures introduced to minimise the transmission of COVID‐19 resulted in restrictions in social contact, and for some, isolation for long periods, with consequences for psychological, social, and physical health for people with dementia (Pongan et al., 2021). These consequences could be particularly significant for people with dementia and their carers, groups for whom social inclusion is particularly important with regard to maintaining well‐being (Greenwood et al., 2018; Pinkert et al., 2021). Under normal circumstances, people with dementia are vulnerable to social isolation due to a reduction in social networks and support (Kane & Cook, 2013; Gardiner et al., 2018). People with dementia and carers are already marginalised (Wright & O'Connor, 2018) and potentially at the risk of social exclusion resulting from individual circumstances, health status, social attitudes, and policy frameworks (Khan et al., 2015; Pinkert et al., 2021). Pandemic‐related restrictions could increase the risk of social exclusion, especially for those with difficulties accessing healthcare and civic amenities (O'Rourke et al., 2021a; Seifert et al., 2021). Among the dimensions of social exclusion commonly experienced by older people (Walsh et al., 2017), three domains are most likely to be exacerbated by the impact of pandemic‐related restrictions: social relations, services, amenities and mobility, and neighbourhood and community.

International evidence has already highlighted the complex difficulties experienced by people with dementia and carers due to social restrictions during the pandemic (Clemente‐Suárez et al., 2021). Early surveys suggest that protracted isolation has triggered an increase in neuropsychiatric symptoms and functional decline for people with dementia (Borelli et al., 2021; Clare et al., 2021; Cohen et al., 2020; Ismail et al., 2021; Simonetti et al., 2020), and qualitative studies have reported worsened cognitive ability, anxiety, and irritability (Tuijt, Frost, et al., 2021). Carers have experienced deterioration in mental and physical health (Alzheimer's Society, 2020; Masterson‐Algar et al., 2021) due to the increased burden of care and lack of access to support or respite (O'Rourke et al., 2021a; Cagnin et al., 2020; Canevelli et al., 2020; Carpinelli Mazzi et al., 2020; Giebel, Lord, et al., 2021). These challenges have affected social relations, placing extra strain on the relationship between the carer and the person with dementia and fuelling feelings of isolation (Tuijt, Frost, et al., 2021), especially in the context of restricted face‐to‐face interaction. At the same time, services, support, and amenities became unavailable with the closure of dementia services, reduced access to healthcare, and shifts to telehealth appointments (O'Rourke et al., 2021a; Kalicki et al., 2021; Tuijt, Rait, et al., 2021).

Several studies conducted earlier in the pandemic have suggested that social distancing measures and the closure of support services have led to feelings of abandonment amongst people with dementia and carers (Bacsu et al., 2021; Clare et al., 2021; Giebel, Lord, et al., 2021; O'Rourke et al., 2021a; Rochford‐Brennan & Keogh, 2020). Feelings of abandonment could indicate that people were experiencing social exclusion. In this study, we set out to explore further the impact of pandemic‐related social restrictions and the extent to which these contributed to a sense of social exclusion, and to examine what might help to prevent or mitigate such effects. We conducted qualitative interviews with people living with dementia and carers during a period of changing social restrictions eight months after the start of the COVID‐19 pandemic in England and Wales, and before the introduction of the vaccination programme.

## 2 METHODS

### 2.1 Design

This is a qualitative interview study using framework analysis to identify themes in participants' accounts (Gale et al., 2013).

### 2.2 Recruitment and data collection

INCLUDE—Identifying and mitigating the individual and dyadic impact of COVID‐19 and life under physical distancing on people with dementia and carers (Clare et al., 2021) is embedded within the longitudinal IDEAL cohort study (Clare et al., 2014; Silarova et al., 2018). INCLUDE is a mixed‐methods study involving a quantitative survey and qualitative interviews. All original IDEAL cohort participants with dementia were community‐dwelling and had mild to moderate dementia at the time of recruitment into the original IDEAL cohort study, and in each case, an informal family carer was invited to participate. IDEAL was approved by Wales Research Ethics Committee 5 (reference 13/WA/0405) and IDEAL‐2 by Wales Research Ethics Committee 5 (reference 18/WS/0111) and Scotland A Research Ethics Committee (reference 18/SS/0037). INCLUDE was approved as an amendment to IDEAL‐2 for England and Wales (18/WS/0111 AM12).

Participants in the INCLUDE study who had already been interviewed using remote methods for the quantitative survey element of the study, who were still living at home and had consented to be contacted for a follow‐up interview (*n* = 35 individuals at the time of recruiting), were eligible. People with dementia and carers, either as individuals or dyads, were invited. We used convenience sampling whilst attempting to capture some diversity (male and female, different dementia types, England and Wales) in the sample. Potential participants received information about the study in their preferred format and were given time to ask questions, and time to consider taking part. Consent was provided by telephone or Zoom, and was recorded by the researcher.

CP and RC (both experienced post‐doctoral researchers working for the IDEAL programme), telephoned 27 people. Six were not contactable and two were not telephoned as we stopped recruiting when the vaccine roll‐out started. Interviews were offered by telephone, Microsoft Teams, or Zoom, according to individual preference, and participants could conduct the interview in one or several shorter interviews according to their wishes.

Participants were recruited between November 4, 2020 and December 1, 2020. In England and Wales, the vaccination programme was expected to start early in December. In England, COVID‐19 infection rates had risen to 664,700 cases in the week ending November 14, and in Wales infection rates had stabilised to 18,400 cases (Office for National Statistics, 2020). On November 5, England moved into the second national ‘lockdown’ with guidance to stay at home except for essential shopping or medical needs, and not mix with another household accept for caring needs. Medically vulnerable people were no longer required to isolate, but people aged over 60 years were advised to minimise social contact. However, across England, restrictions were expected to ease during the December national holidays. In Wales, from November 9, restrictions were eased somewhat. Social venues and non‐essential shops reopened allowing up to four people to meet, and travel restrictions within but not beyond Wales, were lifted.

Interviewers (CP and RC) conducted semi‐structured interviews with no theoretical focus using a topic guide (Table 1) designed with input from the IDEAL Patient and Public Involvement (ALWAYS) group (Litherland et al., 2018). The questions aimed to elicit the experiences of participants from their perspective as someone with dementia, or a carer of someone with dementia. The interviewers asked about the implications of the current restrictions, any changes to routine, any received or missing support over the course of the pandemic, and any expected implications of the vaccine programme.

**TABLE 1:** Topic guide showing topics raised for each of the stages experienced during the pandemic

Timeline	Topic
Start of lockdown	Difficulties or changes to daily routines.
	Own coping strategies and or support found to be helpful.
Some loosening of restrictions 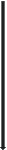	Additional or missing support or information that might have been helpful.
	Any unexpected benefits or outcomes, and how they might be maintained.
The situation now	Training or information health or social care professionals or volunteers need to help people with memory difficulties in the COVID‐19 situation.

### 2.3 Analysis

Two researchers (CP and RC) undertook framework analysis as it offers a systematic methodology to develop themes and is well‐suited to analysis of semi‐structured interviews with a policy focus (Gale et al., 2013).

All interviews were digitally audio‐recorded and transcribed verbatim, anonymised, and uploaded to Nvivo12.

Analysis followed the stages of Framework as set out by Gale et al. (2013). The researchers familiarised themselvs with all interviews by reading the interview notes and transcripts and listening to the interviews. They then independently open‐coded two carer transcripts and two transcripts from people with dementia, and developed the first framework inductively by comparing and revising the codes until agreement was reached. The framework was then applied to the remaining interviews. CP and RC revised the framework together as the interviews were coded. Themes were then developed by organising the codes of the revised framework into categories and themes relevant to answering the research questions. CP then charted the coded data into a matrix to explore differences and similarities among people with dementia and carers, and then tested possible interpretations against the original data. CP and RC discussed the emerging findings with the research team, including RL who discussed the findings with our Patient and Public Involvement Group (Litherland et al., 2018) to explore alternative interpretations.

## 3 FINDINGS

### 3.1 Participants

Two researchers conducted 19 interviews representing 22 people: 12 people with dementia and 10 carers. Two people with dementia and two carers were Welsh, the others were English. Three of the interviews were conducted with dyads interviewed together, and the members of one dyad (PD4 and C4) were interviewed separately. The other 13 interviews were with individuals not connected with any other participant. The mean duration of interviews with people with dementia was 37 min. Carers’ interviews averaged 40 min, and the joint interviews averaged 49 min. The most common dementia diagnosis was Alzheimer's disease (7/12), the ages ranged between 50 and 88 years, and 4/12 lived alone. Carers were aged between 61 and 78 years, all lived with the person with dementia, one was the son and the others were married or in a partnership. Participant characteristics can be found in Tables 2 and 3.

**TABLE 2:** Characteristics of the participants with dementia

ID	Interview duration in minutes	NationalityW = WelshE = English	Age in years	Gender	Dementia type	Living arrangements
PD1	33	E	67	M	AD	Living with partner
PD2	27	W	84	F	AD	Living alone
PD3 J	23	W	88	F	AD	Living with spouse
PD4	35	E	64	M	VaD	Living with partner
PD5	28	E	77	M	AD	Living alone
PD6	24	E	67	M	FTD	Living with partner
PD7	47	E	50	F	AD	Living alone
PD8 J	51	E	75	M	AD	Living with spouse
PD9 J	60	E	71	M	FTD	Living with spouse
PD10	53	E	65	M	Mixed	Living alone
PD11	41	E	70	M	FTP	Living with spouse
PD12	32	E	61	F	AD	Living with spouse

Abbreviations: AD, Alzheimer's disease; FTD, Fronto‐temporal dementia; J, Joint interview with the person with dementia identified by the equivalent study ID; Mixed, Mixed Alzheimer's and vascular dementia; VaD, Vascular dementia.

**TABLE 3:** Characteristics of the participating carers

ID	Interview duration in minutes	NationalityW = WelshE = English	Age in years	Gender	Relationship to person with dementia. All were living with the person with dementia
C1	45	E	70	M	Partner
C2	25	W	65	M	Son
C3 J	23	W	73	M	Spouse
C4	53	E	72	F	Spouse
C5	32	E	61	M	Spouse
C6	51	E	78	F	Spouse
C7	27	E	72	F	Spouse
C8 J	51	E	70	F	Spouse
C9 J	60	E	72	F	Spouse
C10	43	E	74	M	Spouse

Abbreviation: J, Joint interview with the person with dementia identified by the equivalent study ID.

### 3.2 Themes

The topics raised by participants during the interviews are grouped under three main themes. These comprise (a) *fear of decline*—the concern about a loss of skills caused by social distancing measures and efforts to avert decline; (b) frustration concerning the *dilemma of re‐engaging*—not feeling able to return to normal even when restrictions are eased, and (c) *neglect*—disappointment about the lack of concern or consideration from individuals, groups, and authorities.

#### 3.2.1 Fear of decline

There were some people with dementia who had few concerns and enjoyed the quiet time during the pandemic, but the overarching feeling was that there were negative impacts on dementia‐related symptoms and mood, related to not feeling able to engage in usual social and other activities. This incentivised people with dementia to attempt to avert decline and to maintain a positive attitude by being busy. For carers, the additional responsibility and concerns about decline was difficult.

Many people with dementia understood the importance of social contact and the value of keeping busy to maintain social skills and self‐confidence to interact with others. A deterioration in abilities was understood to create difficulties for re‐engaging with society, even when restrictions were eased.

P10 my belief is that it was the dementia was accelerated on the communication side because of lack of practice. I wasn't doing things, I wasn't seeing people, not just the vocal side and the hearing side, but because of not being with people my almost body language reading skills deteriorated as well.

Whilst people with dementia spoke of their concern about their progression of dementia symptoms, some carers reported sadness relating to the changes in the skills and behaviour of the person with dementia and a realisation of the impact the changes had on the relationship. People with dementia did not mention this. Other carers spoke of the positives of doing more activities together and valued a strong relationship that had helped them cope with the pressures of the pandemic. Being alone with the person with dementia was harder for those carers who felt their relationship had changed.

C9 I think it's just companionship, normal conversation and companionship, somebody just to… because [name's] lost his empathy and his interest in things, just somebody for me, do you know what I mean?

A change in the relationship was linked to increased responsibility to manage new risks relating to COVID safety and keeping the person with dementia occupied, but it could be challenging making sure there was enough to do.

C10 Obviously, it meant a bit more thinking ahead. So it was trying to find things that she was interested in. …it was also keeping my wife engaged with it and not trying to do too much…

Although some enjoyed activities that they could do together, many carers felt lonely and bored, with limited opportunities to focus on their own well‐being. As face‐to‐face support groups and services had stopped, both people with dementia and carers were isolated with no options for carer respite.

C4 he would go to the [name of service], which was the day centre; they'd come and pick up, which I paid for obviously, and they dropped him back, but it gave me 6 h in which I could, um, do, you know! Without having to be watchful.

Averting decline and maintaining positive mental health

People with dementia experienced distress over expectations or experience of decline, but wanted to ensure they spent time positively. People with dementia spoke about the importance of doing familiar but also new things. Several wanted to challenge themselves and practise skills they believed would be of benefit. This was purposeful and people with dementia found strength in realising they had been able to adapt and learn. For example, joining online groups, doing mind training, learning names of people or objects when out, reading, and improving recognition of shape and colour.

PD7 because of lockdown, I just got a 500‐piece [jigsaw] but it was like… like brightly coloured and it… although it was hard to do I persevered with it.

PD4 I realised that if I keep my brain active hopefully I can slow the process down of the disease

Being in contact with others was important to maintaining well‐being. One element was altruism. One respondent (PD11) was determined to visit his friend in a care home and to continue to teach nursing students about dementia by using Zoom. This couple valued being National Health Service (NHS) patient representatives:

C8 But of course, because we've both been at home, it's… it's just been a good thing for us. And it's really, having the NHS meetings that's… that's helped us.

PD8 It's helped us survive

For both people with dementia and carers with internet access, online social media was useful in the absence of face‐to‐face contact. This allowed meetings to continue, and in some cases increased contact with loved ones and support networks. However, those who did not have access to the internet may have missed out.

C1 so I've adapted that by ringing people what… when I need, well, ringing people on a regular basis really and so still maintaining contact but not as… not as much if I was on Skype maybe.

A commonly mentioned change during restrictions was increasing exercise levels. Three respondents with dementia talked extensively about their reasons for getting out for walks to manage their mental and physical well‐being. One man enjoyed some chance social interaction whilst exploring his local woods:

PD6 I've met some very nice people, and it's… we were able to sort of have a brief talk and jokes and things like that, but still keeping our two metres apart, that sort of thing.

Another (PD1) enjoyed practising learning new walking routes and memorising names of people he met on his walks, and was also ‘quite proud’ of improving his diabetes control through increased exercise. Another man (P10) was determined to lose weight and improve his mobility but to maintain distance from people, he walked laps around his communal garden at night.

#### 3.2.2 Dilemma of re‐engaging

People with dementia experienced a dilemma in wanting to return to social activities, but worsened dementia symptoms and continuing barriers within society made that hard, even with the easing of social restrictions. The promise of vaccinations did not instil confidence.

PD6 is it ever going to get rid of coronavirus?

They were wary about safety in the case of future easing of restrictions, and many struggled to remember or did not trust the frequently ‐changing official information. Some had found it more comfortable to stay indoors.

PD4 I force myself to go out now. And I think this second lockdown is an excuse for me in a way that I can hide, that I don't want to go out much…

A loss of confidence in memory and remembering the rules were concerns for both people with dementia and carers, and were complicated by a more generalised feeling of anxiety about returning to mixing with people, thus the cyclical problem of prolonging their social isolation.

PD1 I'm very nervous, and still am of going outside the house. It's going into places where other people are. They creep up on you and accumulate round without even thinking. Because it is… it is very easy to… to not social distance when you're out because you tend to forget.

C10 …I'll say, ‘Well it's because of the virus?’ ‘What virus?’ But I just have to be very close to her all the time to make sure she's not doing something that causes a problem.

People felt nervous about going into healthcare settings during the pandemic but felt reassured by appropriate COVID‐19 safety precautions when attending hospital and dental appointments. In contrast, there was some displeasure about the shift to telephone or online appointments. People wanted choice about the delivery of appointments. Some people with dementia wanted the option of having the carer present and a choice of telephone, online, or face‐to‐face appointments (PD10). A carer (C5) described a long wait for a dementia review over the phone, but the person with dementia was contacted at very short notice, which was unsatisfactory because the carer needed to be present.

A change in the usual medical professional seen for dementia‐related appointments caused some upset. People with dementia felt that if the practitioner did not know them, any changes in their dementia‐related needs would not be understood. Also, people with dementia wanted more information about what they could do during COVID‐19 to remain independent and engage in meaningful activities for ‘interaction and stimulation’ (C10), but this was not being offered.

PD10 …references I made to how I thought things had changed were dismissed in saying, “Well, you know, dementia does progress”…I wanted somebody to, kind of, give me coping strategies in a way, you know, almost like if… if it had been a physical thing, they'd give you exercises to do.

When talking about reduced social restrictions, some of the participants were uneasy because they thought members of the public would not understand their difficulties or vulnerability. One carer mentioned her support for a local initiative of wearing a lanyard to identify someone with dementia in health settings, but others had concerns about public perceptions of people with dementia:

C9 I think you've got to be careful because I think once you start putting a label on them, you know, that they've got dementia they're open to abuse, aren't they?

To help people with essential needs, some high street adaptations had been helpful, such as a library providing a pick‐up and drop‐off point and a pharmacy delivering medicines to the car (PD2).

Another barrier encountered was a lack of understanding of the needs of people with dementia and carers when in shared public places.

C10 all the seating that had been provided there, most of it disappeared. And then the small amount of seating that was, was clearly labelled, ‘Only for people with disabilities.’

This man summed up his feelings about the dilemma faced by people with dementia in getting back to normal:

PD10 From a cosmetic point of view it was beneficial for the government to make us fearful so that we would stay in. Well, they've now got to spend millions undoing that…So that we feel like going out again.

#### 3.2.3 Neglect

Not being contacted by individuals, groups, or authorities, not feeling included in official information provided, or in arrangements for easing restrictions, led to a feeling of neglect and exclusion. Two carers suggested that people with dementia were not given due consideration during lockdown in the same way as people with physical disabilities.

C10 It's a bit like obviously if you turn up at the door with someone in a wheelchair, it's obvious that they're disabled… the general public don't have much of an understanding of what difficulties you get with mental health problems, no matter what they are, but particularly with dementia.

Many felt surprised they had not been contacted, for example, from church leaders support services or medical professionals.

C2 I think there should have been some sort of communication to see if she was alright. And they used to come… so I'm a bit disappointed that they haven't done that this time, like, you know?

People did not tend to be proactive in asking for assistance, but wanted to be asked about their well‐being. By this stage in the pandemic, people had found solutions to meet their essential needs, but some felt the typical type of support offered was not what they needed.

C10 It never occurred to me to actually talk to people about it. Because you've then got to specify exactly what you want. I don't need physical support. I don't need any domestic support. But it's interaction and stimulation.

Government information was found to be as confusing and non‐specific to dementia, and so people were not sure which rules were relevant. Carers were also worried that the person they cared for would not be able to follow the distancing or mask‐wearing rules (C10, C4 and C2).

C6 I don't actually know which group we're supposed to be in, and what we're supposed to be doing and not doing.

Two people with dementia suggested there should be a central source for clear and trusted information, and somewhere to go in a time of crisis. Both people with dementia and carers thought that dementia networks were useful sources of information.

C4 [the] memory café is where, er, you tend to find out what's available, who to go to, who to speak to…You know, and you get that sort of thing.

There was also some disappointment about the lack of consultation regarding reopening groups. One man who was caring for his wife with advanced dementia, for whom internet contact was not possible, understood the group could not continue day trips by bus, but had ideas about possible alternatives (such as a socially distanced walk); however, the service had not been in contact about reopening.

C5 Talking to fellow caregivers, I think we would have been far more bold, actually… Because of the benefit, both to me and to [name] I feel that she gets from the group, if they could have found some way of bringing them together.

## 4 DISCUSSION

This study explored the impact of social distancing measures on people with dementia and carers living in the community during restrictions and before the COVID‐19 vaccination roll‐out in England and Wales. We identified three interrelated experiential themes that add to the existing literature focussing on earlier experiences of the pandemic: fear of decline, the dilemma of re‐engaging, and neglect. Many of the interviewees found ways to adapt their behaviour to stay positive and help prevent a decline in capabilities or mood, but some experienced reduced confidence with a decline in capabilities. This created a cyclical dilemma that made returning to usual activities difficult even during periods of reduced restrictions. Re‐engaging with society was challenging due to feeling less capable, or feeling that different aspects of society had not adjusted adequately to be inclusive. Knowledge of the upcoming vaccination programme did not instil feelings of hope. These experiences related to several domains relevant to social exclusion (Walsh et al., 2017), in particular services, amenities and mobility, social relations, and community. The impact of this, although relevant to the older population, was exacerbated by the challenges of living with dementia or caring for a person with dementia.

In this study, cultural, social, and emotional dimensions of exclusion impacted heavily on both people with dementia and carers during the pandemic because meaningful social connection both at a personal and a civic level was important for self‐worth and stimulation to help avert dementia progression. There was less evidence to show that people felt excluded at an economic or environmental level. These findings support evidence that the quality of relationships and opportunities to socialise are pivotal elements of social inclusion for people with dementia (Pinkert et al., 2021. Quinn, Hart, et al., 2021, Quinn et al., 2021). Social relations are a dimension of social exclusion in older age (Walsh et al., 2017), but the reduction in or loss of skills in communication during the pandemic for people with dementia could lead to further isolation and restriction of opportunities for social relations and civic engagement. In addition, for carers, increased practical caring responsibilities and dealing with behavioural issues are known to impact the relationship with the care recipient (Quinn et al., 2009), potentially furthering the experience of isolation. Others have found the additional stress caused by having to balance COVID risk with well‐being added to carers' responsibilities (Cagnin et al., 2020). In our study, in some cases, the relationship between the carer and the person with dementia seemed to be strengthened by spending more time together, but for others, the relationship with the person with dementia changed with a rapid increase in responsibility and care needs.

The experience of decline in abilities for the person with dementia and greater caring responsibilities for carers led to increased vulnerability to being excluded. Increased responsibility meant reduced opportunities for carers to have social interaction with others, or to have respite when things reopened. For people with dementia, loss of communication skills and confidence made interaction with friends and family, and the wider community, more problematic and possibly irretrievable. Experiences, or fears of stigma reported widely pre‐pandemic (Herrmann et al., 2018), were alluded to and are likely to have been worsened due to reduced capabilities or fears about loss of capabilities.

Both people with dementia and carers as individuals or dyads, felt they were left to deal with the consequences of the immediate or longer‐term implications of the pandemic but found their own solutions to manage as best as they could, often changing behaviours and adapting. Although personal strategies were useful, and some may have felt comfortable with reduced social contact, there was little evidence of specific dementia support at a broader social or cultural level. There was little evidence of the experience of decline being acknowledged or understood outside of individual experience, indicating social exclusion at the meso‐ (interactional environment) and macro‐ (broader social) levels (Pinkert et al., 2021).

The dilemma of people with dementia and carers wanting to re‐engage with society but being restricted by changes in dementia symptoms and feelings of increased vulnerability adds understanding to the ‘complex health issues’ (Pinkert et al., 2021) causing micro‐level social exclusion. Both people with dementia and carers experienced exclusion via a lack of consultation about returning to groups or services. Official COVID‐19 information was neither clear nor specific to their situation. The consequences of reduced social interaction have been reported by both people with dementia and carers elsewhere (Giebel et al., 2020; O'Rourke et al., 2021b; Talbot & Briggs, 2021), and are anecdotally supported in online forums such as www.dementiadiaries.org but our data show this was not addressed as the pandemic progressed.

The overarching feeling of neglect amongst people with dementia and carers during the pandemic was identified in our earlier study conducted in the first wave of the pandemic (O'Rourke et al., 2021b). There is a wider and ongoing problem of social exclusion affecting people with dementia, but this has almost doubled during the pandemic (Cohen et al., 2020), and the lack of hope to move forward out of the pandemic is a cause for concern. A general feeling of needs not being understood could have contributed to low expectations based on experience. To address the consequences of pandemic restrictions, and possibly to gain control, individuals adapted to coping, a process also seen previously (O'Rourke et al., 2021b). Accessing online social activities, groups, and information was a source of comfort for some, but also a source of exclusion for those unable to participate. Where inclusion was promoted this was demonstrated through individual examples of localised access to goods and services. These local adaptations were not dementia‐specific but helped people to go about their usual activities, and may have helped them feel included.

It is important to recognise the limitations of the study. We recruited participants from a relatively small number of willing individuals who were able to engage in interviews using remote methods. Although this facilitated timely completion of this ‘rapid response’ COVID study, the potential to recruit over a longer period of time might have resulted in a larger number and may have uncovered other exclusionary impacts of COVID restrictions. Also, as we were only able to recruit people who could use the telephone or the internet, we may have excluded people with more advanced dementia. Despite the limitations, the main strength of this study is that the findings deepen understanding of the consequences of ongoing cycles of social restrictions in addition to the pre‐existing risk of social exclusion amongst people with dementia and has implications for a return to usual social activities. This study offers some insights into the ongoing needs of both people with dementia and carers despite the promise of vaccinations.

People with dementia and carers identified what was important to them and attempted to mitigate the impacts of social isolation, but more help was needed. There was a lack of proactive contact with people with dementia from health and social care, or council‐based services, and although some ‘checking‐in services’ to see how people were, and to signpost to support, were appreciated earlier on in the pandemic (O'Rourke et al., 2021b), these did not continue for our participants. While much of society had enjoyed the easing of restrictions at various stages over the preceding summer months, our group had not felt able to re‐engage in the same way as other members of the public. In the context of moving to post‐pandemic ‘recovery’ with more people vaccinated, a focus on better respite for carers, and on rebuilding confidence and rehabilitation for people with dementia, may be worthwhile. The difficulties in accessing or using the internet to find information and services should be acknowledged and alternative methods for keeping people in contact and informed could be explored. There is a need to offer a range of options for accessing health and social care rather than relying too heavily on telephone‐ or internet‐based appointments for people who may find these forms of communication difficult (Kalicki et al., 2021); the shift to online groups and services was initially welcomed but the longer‐term effects are not yet evaluated. Alongside this, governments and policy makers could do more to include the voices of people with dementia in finding ways of allowing them to feel safe accessing the shops, services, and entertainment that normally form part of their lives.

## 5 CONCLUSION

Qualitative data collected during the pandemic has uncovered the need for people with dementia and carers to feel part of normal society during periods of severe social restrictions, and this study offers some solutions for the future. Although people demonstrated problem‐solving ability and resilience, the pandemic appeared to have exacerbated the extent of social exclusion, and there was little expectation of external intervention. People with dementia and their carers needed practical advice about what they could do during the pandemic to stay actively and meaningfully engaged, and carers experiencing additional demands needed practical solutions to enable them to gain respite. These findings offer guidance about supporting people with dementia and carers during future periods of pandemic‐related social restriction.

## AUTHOR CONTRIBUTIONS

CP conducted interviews, carried out the analysis, devised and wrote the draft paper. LC devised the study, provided critical revision of the article and agreed upon the final version to be published. RC conducted analysis and interpretation, took part in ongoing discussions about analysis and findings, provided critical revision of the article, and approved the final version to be published. SS, CV, CQ, AH, RL, LA took part in ongoing discussions about analysis and findings, provided critical revision of the article, and agreed upon the final version to be published.

## FUNDING INFORMATIONS

‘Identifying and mitigating the individual and dyadic impact of COVID‐19 and life under physical distancing on people with dementia and carers (INCLUDE)’ was funded by the Economic and Social Research Council (ESRC) through grant ES/V004964/1. Investigators: Clare, L., Victor, C., Matthews, F., Quinn, C., Hillman, A., Burns, A., Allan, L., Litherland, R., Martyr, A., Collins, R., & Pentecost, C. ESRC is part of UK Research and Innovation (UKRI). INCLUDE data will be deposited with the UK data archive in June 2022 and will be available to access from June 2023. ‘Improving the experience of Dementia and Enhancing Active Life: living well with dementia. The IDEAL study’ was funded jointly by the Economic and Social Research Council (ESRC) and the National Institute for Health and Care Research (NIHR) through grant ES/L001853/2. Investigators: L. Clare, I.R. Jones, C. Victor, J.V. Hindle, R.W. Jones, M. Knapp, M. Kopelman, R. Litherland, A. Martyr, F.E. Matthews, R.G. Morris, S.M. Nelis, J.A. Pickett, C. Quinn, J. Rusted, J. Thom. ESRC is part of UK Research and Innovation (UKRI). IDEAL data were deposited with the UK data archive in April 2020, and will be available to access from April 2023. Details of how the data can be accessed after that date can be found here: http://reshare.ukdataservice.ac.uk/854293/ ‘Improving the experience of Dementia and Enhancing Active Life: a longitudinal perspective on living well with dementia. The IDEAL‐2 study’ is funded by Alzheimer's Society, grant number 348, AS‐PR2‐16‐001. Investigators: L. Clare, I.R. Jones, C. Victor, C. Ballard, A. Hillman, J.V. Hindle, J. Hughes, R.W. Jones, M. Knapp, R. Litherland, A. Martyr, F.E. Matthews, R.G. Morris, S.M. Nelis, C. Quinn, J. Rusted. L. Clare and L Allan acknowledge support from the NIHR Applied Research Collaboration South‐West Peninsula. The views expressed are those of the author(s) and not necessarily those of the ESRC, UKRI, NIHR, the Department of Health and Social Care, the NHS, or Alzheimer's Society. The support of ESRC, NIHR, and Alzheimer's Society is gratefully acknowledged.

## CONFLICT OF INTEREST

We have no conflict of interest to declare.

## Data Availability

IDEAL data were deposited with the UK data archive in April 2020 and will be available to access from April 2023. Details of how the data can be accessed after that date can be found here: http://reshare.ukdataservice.ac.uk/854293/ INCLUDE data will be deposited with the UK data archive in June 2022 and will be available to access from June 2023.
